# Therapeutic Effects of 17β-Estradiol on Pelvic Organ Prolapse by Inhibiting Mfn2 Expression: An *In Vitro* Study

**DOI:** 10.3389/fendo.2020.586242

**Published:** 2020-11-25

**Authors:** Xiao-Qing Wang, Rui-Ju He, Bing-Bing Xiao, Ye Lu

**Affiliations:** Department of Obstetrics and Gynecology, Peking University First Hospital, Beijing, China

**Keywords:** pelvic organ prolapse, 17β-estradiol, Mitofusin2, uterosacral ligament fibroblasts, procollagen

## Abstract

**Objective:**

To assess the effects of 17β-estradiol (E2) on proliferation, apoptosis, and protein expressions of fibroblasts at different concentrations and time intervals to reveal the mechanism of E2 in the treatment of pelvic organ prolapse (POP).

**Study Design:**

The uterosacral ligament fibroblasts were collected from seven POP patients for primary culture of fibroblasts. The culture media containing 0, 10^-6^, 10^-7^, 10^-8^, and 10^-9^ mol/L E2 were used for 24, 48, 72, and 96 h.

**Main Outcome Measures:**

The cells were collected for cell counting kit-8 (CCK-8), apoptosis, quantitative reverse transcription polymerase chain reaction (qRT-PCR), and Western blotting assays.

**Results:**

Compared with the control group, in the values of fibroblasts cultured in 10^-8^ mol/L E2 for 72 h, the proliferation, mRNA and protein expression of Mitofusin-2 (Mfn2) separately increased (P < 0.05), decreased (P<0.001) and decreased (P<0.001). However, the expression level of procollagen 1A1/1A2/3A1 and cyclinD1 markedly increased (P<0.001, all), which was consistent with the results of protein level. What’s more, the expression of estrogen receptor α(ERα), estrogen receptor β(ERβ) and G protein-coupled receptor 30(GPR30) were significantly increased in 10^-8^ mol/L E2 group.

**Conclusions:**

E2 can inhibit the progress of POP by inhibiting the expression level of Mfn2, as well as promoting expression of procollagens and proliferation of fibroblasts. This effect is time- and concentration-dependent. Only when the estrogen concentration reaches 10^-8^ mol/L, the therapeutic effect is the greatest after 72 h.

## Introduction

Pelvic Organ Prolapse (POP) seriously affects women’s quality of life. The symptom that most strongly correlates with POP is a vaginal bulge that can be seen or felt (1). It is now established and widely accepted that the decreased collagen fiber synthesis or increased degradation in pelvic floor tissue leads to POP (1–3). The most effective treatment method is surgery (1, 3). Estrogen can be used in the clinical treatment of POP (4, 5), making the vaginal mucosa thicker, as well as improving local blood supply, while the specific molecular mechanism has still remained elusive. It has been reported that the quality of vaginal tissue can be improved by placing a continuous low-dose estradiol releasing vaginal ring immediately after pelvic floor reconstruction (4). Local estrogen therapy can increase the blood flow to the genitourinary area and increase the lubrication of vagina (6, 7). However, the evidence-based medical data and mechanism of estrogen treatment for POP are still insufficient (5). Therefore, the present study aimed to reveal the possible role of estrogen in the treatment of POP.

Mitochondria are highly dynamic organelles. They constantly migrate, fuse, and divide to regulate their shape, size, number, and biological function. Mitofusn2 (Mfn2) is a key regulator of mitochondrial fusion and division. It affects cell metabolism, function, proliferation and apoptosis by regulating a variety of signal pathways (8–11). Previous studies on Mfn2 mostly focused on pulmonary fibrosis (10), breast cancer (9), liver fibrosis (10), and Alzheimer’s disease (11), and there was little research on pelvic floor dysfunction. To our knowledge, POP is an aging-related disease, and its relationship with the aging related protein, Mfn2 (12–14), has been rarely reported. However, our previous study (15–17) showed that the expression level of Mfn2 increased, while the expression of procollagen decreased in uterosacral ligament fibroblasts of POP patients. The increased expression level of Mfn2 could inhibit the proliferation and cell cycle of fibroblasts by mediating Ras/Raf/ERK pathway, and led to the decrease of collagen synthesis and secretion, eventually resulting in prolapse. On the basis of previous studies, we, in the current research, attempted to assess the effects of estrogen on proliferation and apoptosis of uterosacral ligament fibroblasts, Mfn2, and procollagen 1A1/1A2/3A1 in POP patients by setting different concentrations and time gradients of estrogen, and also indicate whether estrogen can affect the occurrence and development of POP *via* influencing the expression level of Mfn2.

## Materials and Methods

### Study Subjects

From October to December, 2017, the uterosacral ligament tissues of seven POP patients were collected with the help of the Department of Gynecology and Obstetrics, the First Hospital of Peking University, Beijing, China. The baseline characteristics of 7 patients were as follows: Pelvic Organ Prolapse Quantification (POP-Q) stages (14) II-IV, age of 67.43 ± 7.55 (range, 57.00–77.00) years old, body mass index (BMI) of 23.57 ± 3.33 (range, 17.00–26.30), gravidity at 3.14 ± 1.35 (range, 2.00–5.00), parity 2.14 ± 1.69 (1, 3), and menopausal period of 18.00 ± 7.11 (range, 8.00–25.00) days. The uterosacral ligament was retained for primary culture. All patients had no urinary tract infection, history of vaginal surgery, and/or diseases affecting collagen metabolism, and had not taken 17β-estradiol (E2) within 3 months. The specimens were obtained with the informed consent of the patient. The study was approved by the Ethics Committee of Peking University First Hospital Approval No. 2016(1173).

### Main Reagents and Instruments

Dimethyl sulfoxide (DMSO, A3672) was purchased from AppliChem GmbH (Darmstadt, Germany). TRIzol reagent and reverse transcription polymerase chain reaction polymer kit (AQ131-02) were purchased from Beijing Full-Type Gold Biotechnology Co., Ltd. (Beijing, China); besides, DMEM/F12 (11330-032), antibiotics-antimycotic (15240-062), fetal bovine serum (FBS; 10099141), 0.25% trypsin-EDTA digestive juices (25200-056) were purchased from Gibco (New York, NY, USA). The cell counting kit-8 (CCK-8) was provided by Dojindo Molecular Technologies, Inc. (Rockville, MD USA). Mfn2 monoclonal antibody (dilution, 1/1000; Ab56889) was purchased from Abcam (Cambridge, UK). Anti-procollagen 1A1/1A2/3A1 antibodies (dilution, 1/200; Sc-293182, Sc-166572, and Sc-166333, respectively) were purchased from Santa Cruz Biotechnology Inc. (Dallas, Texas, USA). Anti-ERα (D8H8) Rabbit mAb antibody(dilution, 1/1000) and Anti-cyclinD1 (92G2) Rabbit mAb antibody(dilution, 1/1000) were purchased from Cell Signaling Technology, Inc. (USA). Horseradish peroxidase (HRP) goat anti-mouse IgG (dilution, 1/5000, ab6789), Anti-ERβ(dilution, 1/1000, ab196787) and Anti-GPR30 (dilution, 1/1000, ab39742) were purchased from Abcam (Cambridge, UK). Procedural cooling box was purchased from Sigma-Aldrich (St. Louis, MO, USA).

### Primary Cell Culture, Subculture, and Cryopreservation

During hysterectomy, the fresh uterosacral ligament tissues were taken with the size of 0.5×0.5×0.5 cm^3^. The tissues were washed with phosphate-buffered saline (PBS) containing 1% antibiotics, and the tissues were cut into slices with a diameter of less than 0.1 cm, which were evenly distributed to the bottom of the 25 cm^2^ bottle. Herein, upside-down containers were used and the bottle was carefully turned over after 4–6 h to avoid floating tissue blocks. The medium was prepared with 20% FBS, 69% DMEM/F12, and 1% antibiotics. Besides, passaging the cells was performed after about 15 days. When the cells grew to 70%–80% confluence, the digestion of the cells was conducted with 2.5% trypsin for about 1–2 min, and the reactions were terminated by aspirating the medium. The samples were placed in fridge/cool storage in boxes at -80°C.

### E2 Dissolves Configurations and Stimulates Cells

First, E2 was dissolved with anhydrous alcohol in 20 ug/ml mother liquor, and stored at -20˚C. The cells were then incubated with the concentrations of 0, 10^-6^, 10^-7^, 10^-8^, and 10^-9^ mol/L of serum-free Dulbecco’s modified Eagle’s medium (DMEM), respectively. When the cells were grown to 2 ~ 4 generations, they were collected after 0, 24, 48, 72 and 96h of treating with concentrations of 0, 10^-6^, 10^-7^, 10^-8^, and 10^-9^ mol/L E2.

### Cell Proliferation

Cell proliferation was detected after 0, 24, 48, 72 and 96h of stimulation by E2. After addition of 10 ul CCK-8 detection solution to each well, cells were cultured for 1.5 h, and the optical density (OD) value was measured at the wavelength of 450 nm using a microplate analyzer. Absolute cell numbers were calculated according to a previously prepared standard curve.

### Apoptosis

The rates of cell apoptosis were detected by acridine orange/ethidium bromide (AO/EB) staining after 0, 24, 48, 72 and 96h of E2 stimulation. AO/EB fluorescent staining was carried out by mixing AO (100 ug/ml) and EB (100 ug/ml). After washing thrice with PBS, AO/EB working solution was added. After incubation at 37°C for 30 min, the cells were washed with PBS again for 3 times. The whole process was carried out in the dark condition. The samples stained with fluorescent dyes are observed with a fluorescence microscope. Five fields were randomly selected from each sample, and apoptotic cells were counted separately for subsequent analyses. Cells treated with E2 were digested and collected without EDTA trypsin, washed twice with PBS, centrifuged at 2000 rpm for 5 min, and 5×10^5 cells were collected per sample. The cells were re-suspended in binding buffer, and then, the cells were stained with PE-Annexin V and 7AAD for 10 min at room temperature. The data were analyzed by FlowJo software.

### The mRNA Expression Levels of Mfn2 and Procollagen

Total RNA was extracted by TRIzol reagent after 0, 24, 48, 72 and 96h of stimulation with E2. In each case 1 µg of total RNA was retro-transcribed *via* TransScript One-Step gDNA Removal and cDNA Synthesis SuperMix which contained DNase (AT311-03, TransGen Biotech, China) and 0.05 µg of total RNA was used for the follow-up quantitative reverse transcription polymerase chain reaction (RT-qPCR). Herein, we detected the expression levels of Mfn2, procollagen 1A1/1A2/3A1 and β-actin (housekeeping gene) using RT-qPCR. The relative expressions of genes were calculated using 2 ^- ΔΔCT^. The primers were as follows: Mfn2: forward: 5’-CATCAGCTACACTGGCTCCAACT-3’; reverse 5’-GATGAGCAAAGGTCCCAGACA-3’; Procollagen1A1: forward: 5’-CGAGGGCCAAGACGAAGA-3’; reverse: 5’-CACGTCTCGGTCATGGTACCT-3’; Procollagen1A2: forward: 5’-TGGATACGCGGACTTTGTTG-3’; reverse: 5’-GGCTGGGCCCTTTCTTACAG-3’; Procollagen3A1:forward: 5’-TCGCCCTCCTAATGGTCAAG-3’; reverse: 5’-GGTCACCATTTCTCCCAGGAA-3’; ESR1(ERα):forward: 5’ - ACTTGCTCTTGGACAGGAACC-3’; reverse: 5’- TTCAGGGTGCTGGACAGAAA-3’; ESR2(ERβ): forward: 5’ -TGCTCCCACTTAGAGGTCAC-3’; reverse: 5’- GAAAAGATCA CAAGCGACTTAACG-3’; GPR30: forward: 5’ - ACGAGACTGTGAAATCCGCA-3’; reverse: 5’- CTCTCTGGGTACCTGCCGTC-3’; cyclinD1: forward 5’-CAATGACCCCGCACGATTTC-3’, reverse5’-CATGGAGGGCGGATTGGAA-3’; β-actin forward: 5’-CACGGCTGCTTCCAGCTC-3’; reverse: 5’-CACAGGACTCCATGCCCAG-3’.

### The Protein Expression of Mfn2 and Procollagen in Fibroblasts

The total protein was extracted by radioimmunoprecipitation assay (RIPA), and the protein concentration was determined by the bicinchoninic acid (BCA) assay. The protein samples with known concentrations were added in a predetermined order, with 20 ug protein per well. The proteins were separated by sodium dodecyl sulfate polyacrylamide gel electrophoresis (SDS-PAGE) and transferred onto a polyvinylidene difluoride (PVDF) membrane. The membrane was blocked with 5% milk in Tris-Buffered Saline and Tween 20 (TBST) and incubated with primary antibodies overnight at 4°C. Next, the membrane was washed thrice with TBST and incubated with a HRP goat anti-mouse IgG secondary antibody (dilution, 1:5,000) at room temperature for 1 h. Membranes were washed three times, and enhanced chemiluminescence (ECL) was used for visualizing the proteins of interest as marked by HRP. Image J software was used for quantifications of Western blots bands.

### Statistical Analysis

All data were statistically analyzed by SPSS 23.0 software (BM, Armonk, NY, USA). The patients’ age, BMI, and other measured data were represented by mean ± standard deviation (SD). Normally distributed data were compared by one-way analysis of variance (ANOVA) or independent-sample t-test. K-W test or one-way ANOVA was applied for comparison non-normal distributed data. P<0.05 was considered statistically significant.

## Results

### Primary Culture of Fibroblasts

**Figure 1:**
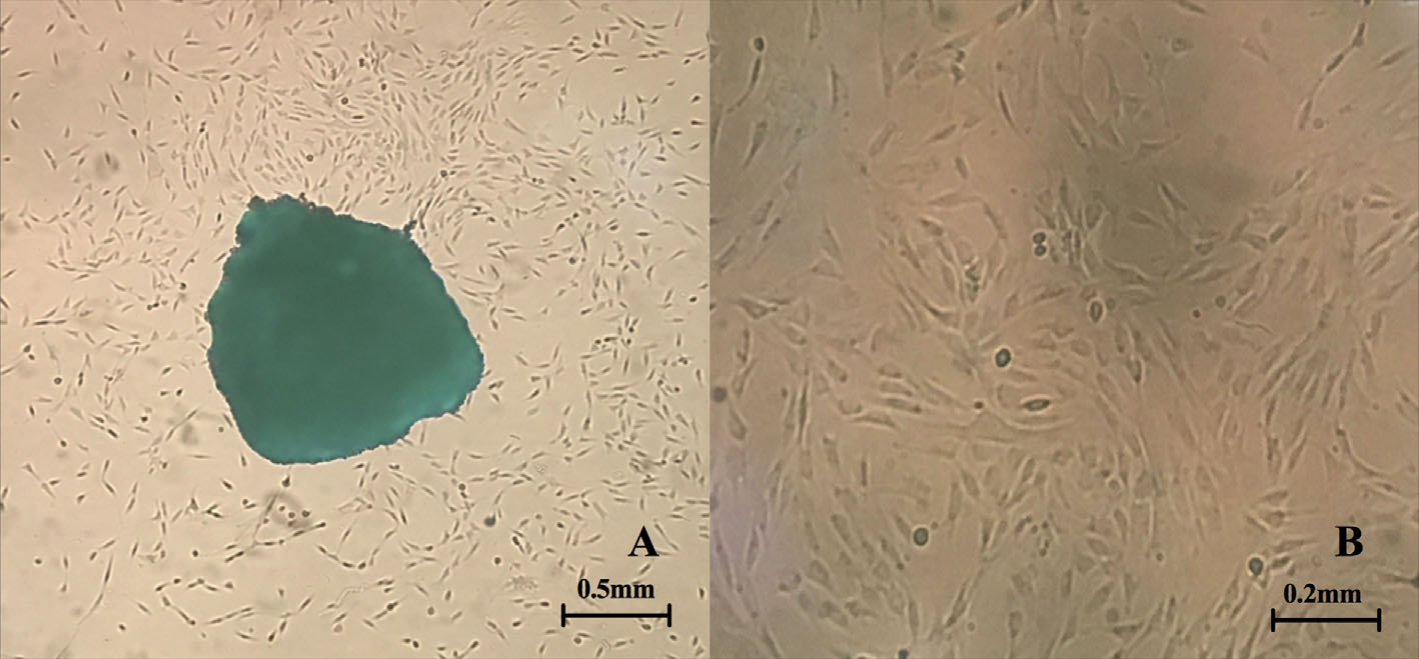
Primary culture of fibroblasts. **(A)** Fibroblasts crawl out of the tissue fragments (the clumps can be observed in the center of the picture) (4X inverted microscope) **(B)** Morphology of fibroblasts after passage (10X inverted microscope).

After inoculation into the culture bottle, a small number of cells were observed, and the cells locally fused into a piece in the tissue block after around 7–10 days, which almost covered areas of the bottle bottom. After about 15 days, the cells were fully fused approximately. The cells were elongated and spindle-shaped or polygonal (**Figure 1**). The growth rate of cells was accelerated after passage, and the cells were then transferred for 3–5 days. The cells were in relatively satisfactory condition within 10 generations.

### Proliferation of Fibroblasts After E2 Stimulation

**Figure 2:**
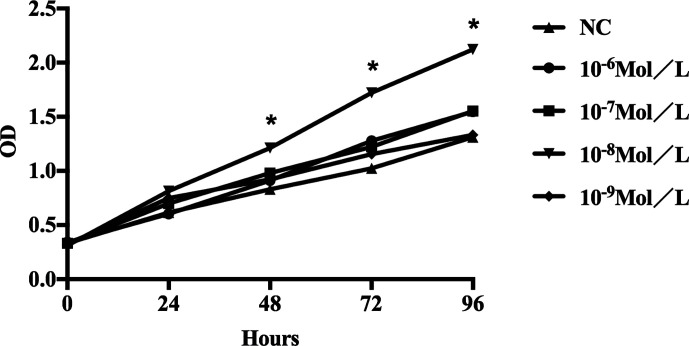
Proliferation of sacral ligament fibroblasts in POP patients stimulated by E2 at different concentrations. The proliferation of fibroblasts increased after E2 stimulation, especially after addition of 10^-8^ mol/L E2, and the proliferation rate of the cells was significantly different from that of the non-E2 group (*P < 0.05).

It was noted that proliferation of fibroblasts increased after E2 stimulation, especially after addition of 10^-8^ mol/L E2, and the proliferation rate of the cells was significantly different from that of the non-E2 group (**Figure 2**). In the NC group(non-treated cells) and the E2 group with concentrations of 10^-6^, 10^-7^, 10^-8^, and 10^-9^ mol/L, the mean optical density (OD) values at hours 0(0 h), 24, 48, 72, and 96 were as follows: (0 h: 0.333, 0.343, 0.332, 0.312, and 0.324); (24 h: 0.615, 0.604, 0.699, 0.814, and 0.749); (48 h: 0.831, 0.911, 0.980, 1.212, and 0.921); (72 h: 1.025, 1.278, 1.222, 1.725, and 1.156); and (96 h: 1.312, 1.547, 1.554, 2.121, and 1.333). The differences were statistically significant. This indicated that 10^-8^ mol/L E2 had the most significant effect on the proliferation of POP fibroblasts. Therefore, we focused on this concentration of E2.

### Apoptosis of Fibroblasts After E2 Stimulation

**Figure 3:**
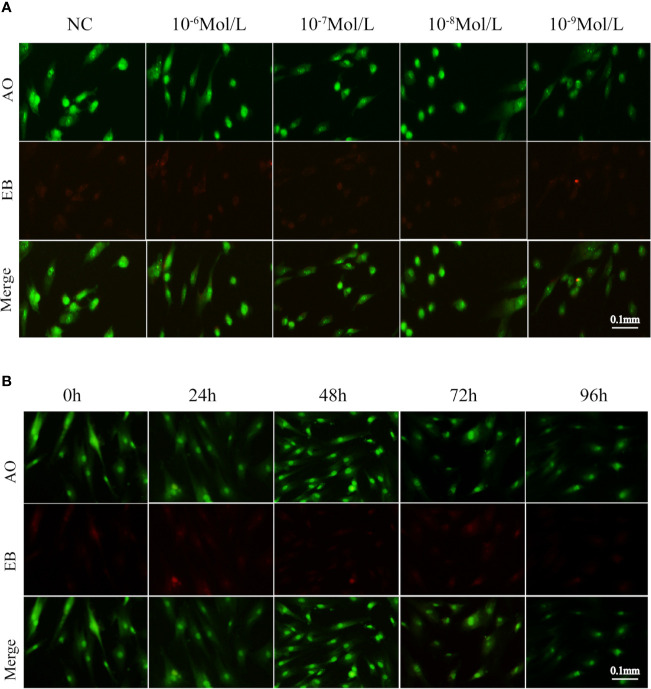
Apoptosis of fibroblasts treated with E2 for 72 h at different concentrations. **(A)** Concentration gradient result of AO/EB after the stimulation for 72h. **(B)** Time gradient result of AO/EB with 10^-8^ mol/L E2. Late apoptotic cells observed in orange color and dead cells(red) were rare among different groups. Normal cells appeared green. There was no statistical difference between early apoptotic cells observed in greenish yellow color treating with concentrations of 0, 10^-6^, 10^-7^, 10^-8^, and 10^-9^ mol/L E2 for 72 h. NC: E2 concentration was 0. Acridine orange (AO): an acridine orange that penetrates the intact cell membrane and inserts nuclear DNA into the cell, causing emission of a bright green fluorescence. Ethidium bromide (EB) can only penetrate cells with damaged membranes, embed nuclear DNA, and emit orange fluorescence.

**Figure 4:**
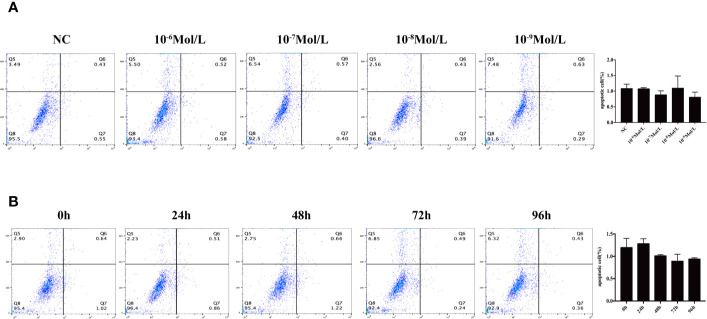
Flow cytometry to detect apoptosis. **(A)** Concentration gradient experiment after the stimulation for 72 h. **(B)** Time gradient experiment with 10^-8^ mol/L E2 Q5: percentage of dead cells, Q6: early apoptotic cells, Q7: late apoptotic cells, and Q8: normal living cells. There was no statistical difference between different groups.

After 24, 48, 72, and 96 h of E2 stimulation, there was no significant difference in the number of apoptotic cells in each group treating with concentrations of 0, 10^-6^, 10^-7^, 10^-8^, and 10^-9^ mol/L E2. The AO penetrates the intact cell membrane and inserts nuclear DNA into the cell, causing emission of a bright green fluorescence. The EB can only penetrate cells with damaged membranes, embed nuclear DNA in agar, and emit orange fluorescence (18–20). Among them, normal cells appeared green. Late apoptotic cells observed in orange color and dead cells appeared red were extremely rare. There was no statistical difference in the number of early apoptotic cells between different groups of E2 whose nuclei were greenish yellow color and presented as dense plaques or fragments. For instance, after 72 h of stimulation (0, 10^-6^, 10^-7^, 10^-8^, and 10^-9^ mol/L E2), the apoptosis was shown in **Figures 3A** and **4A**. In the meantime, the differences in fibroblast apoptosis (10^-8^ mol/L E2) among 0, 24, 48, 72, and 96 h were shown in **Figures 3B** and **4B**.

### RT-qPCR After E2 Stimulation

**Figure 5:**
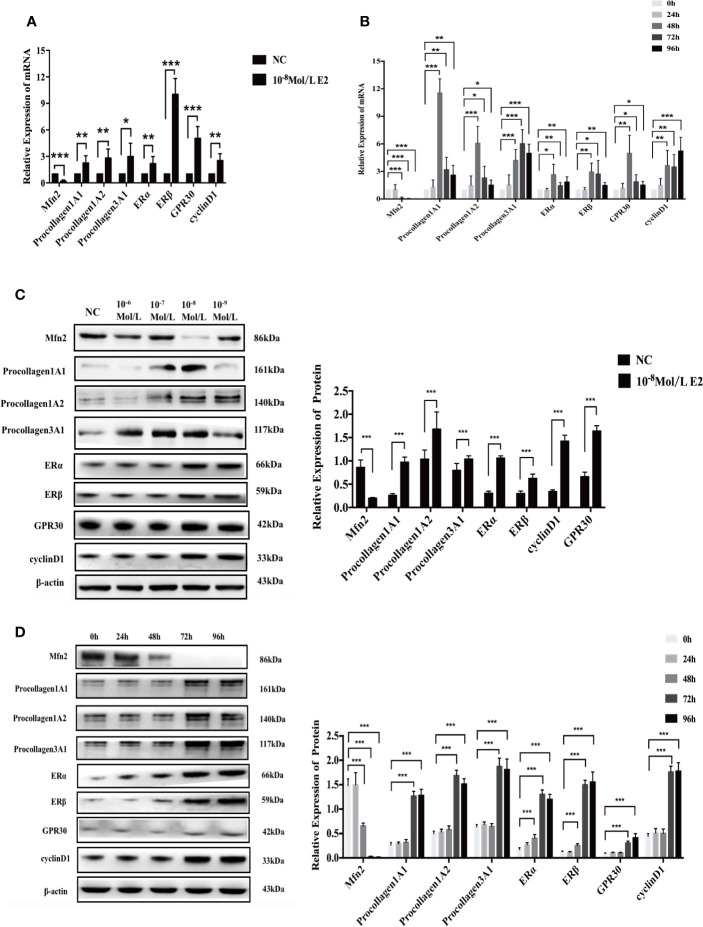
mRNA and protein expression after stimulation of 10^-8^ mol/L E2. **(A, B)** After E2 stimulation of 10^-8^ mol/L for 72 and 96 h, the mRNA level of Mfn2 was decreased, while the mRNA level of procollagen 1A1/1A2/3A1, ERα, ERβ, GPR30, and cyclinD1 were increased. **(C, D)** When uterosacral ligament fibroblasts in POP patients were stimulated by E2 at concentration of 10^-8^ mol/L for 72 and 96 h, the protein level of Mfn2 significantly decreased, whereas the expression of procollagen1A1/1A2/3A1, ERα, ERβ, GPR30, and cyclinD1 were gradually increased. (*P < 0.05, **P < 0.01, ***P < 0.001).

According to the previous results, the groups affected by 10^-8^ mol/L E2 were selected for intensive observation. After 48 h of treatment, compared with the NC group, the mRNA level of Mfn2 remarkably reduced (0.194, P = 0.000), and the expression levels of procollagen 1A1 (2.252, P = 0.007), 1A2 (2.813, P = 0.003), 3A1 (2.990, P=0.012), ERα(2.177, P=0.008), ERβ(10.037, P=0.000), GPR30(5.041, P=0.000), and cyclinD1(2.526, P=0.002) were noticeably elevated in E2 group with concentration of 10^-8^ mol/L (**Figure 5A**).

At the same time, we did a time gradient experiment under the stimulation of 10^-8^ mol/L E2 (**Figure 5B**). Compared with the results of 0 h, the results of Mfn2, procollagen1A1/1A2/3A1, ERα, ERβ, GPR30 and cyclinD1 in 48, 72, and 96 h groups were as follows: (48 h: 0.098; 11.549; 6.079; 4.216; 2.661; 2.957; 4.988; 3.654); (72 h: 0.049; 3.199; 2.304; 6.042; 1.463; 2.723; 1.882; 3.505); (96 h: 0.022; 2.617; 1.520; 4.988; 1.852; 1.487; 1.525; 5.231).

### Western Blotting After E2 Stimulation

Similarly, we first did a concentration gradient experiment (**Figure 5C**). We found that compared with NC group, the protein level of Mfn2 significantly reduced (0.201, P=0.000), and the protein levels of procollagen 1A1 (0.974, P=0.000), 1A2 (1.683, P=0.000), 3A1 (1.041, P=0.000), ERα(1.061, P=0.000), ERβ(0.626, P=0.000), GPR30(1.425, P=0.000), and cyclinD1(1.644, P=0.000) were markedly increased.

In **Figure 5D**, we did a time gradient experiment under the stimulation of 10^-8^ mol/L E2. Compared with the results of 0 h, the changes of Mfn2, procollagen1A1/1A2/3A1, ERα, ERβ, GPR30, and cyclinD1 in 72 and 96 h groups were the most significant: 72 h (0.028; 1.268; 1.692; 1.876; 1.306; 1.502; 0.308; 1.763; P=0.000, all), 96 h(0.017; 1.284; 1.516; 1.814; 1.204; 1.558; 0.415; 1.780; P=0.000, all). These result were consistent with those achieved by RT-qPCR.

## Discussion

Previous studies demonstrated that the majority of patients with POP are postmenopausal women who have low estrogen levels (12–14). A research showed a strong negative correlation between serum estrogen levels and POP-Q scores in patients with POP (21). Therefore, understanding the effects of estrogen on POP, especially the molecular mechanism, is highly essential for prevention and treatment of POP.

In the present study, after culturing the uterosacral ligament fibroblasts and stimulating them with different concentrations of estrogen, we found that compared with medium with concentrations of 0, 10^-6^, 10^-7^, and 10^-9^ mol/L E2, 10^-8^ mol/L E2 medium had more significant effect on inhibiting Mfn2 expression, promoting fibroblast proliferation and procollagen synthesis. At the same time, under the effect of 10^-8^ mol/L E2, Mfn2 expression was significantly inhibited and fibroblast proliferation and procollagen1A1/1A2/3A1 synthesis were significantly increased by 72 and 96 h treatment. What’s more, the expression of ERα, ERβ and GPR30 were significantly increased in 10^-8^ mol/L E2 group.

After E2 stimulation, the proliferation of fibroblasts markedly elevated. Previous study (22) has found that E2 could promote cell growth by up-regulating the expression of cyclinD1, which was related to cell proliferation. This was consistent with the results of our study. Meanwhile, the protein and mRNA expressions of procollagen 1A1/1A2/3A1 significantly increased, which was closely related to POP. A number of scholars demonstrated that supportive structure of pelvic floor is mainly uterosacral ligament, and the change of collagen fiber content in ligament has a great influence on supportive function of pelvic floor (23). Types I and III collagen are the major constituents of collagen fibers, with type I collagen accounting for approximately 90% and type III accounting for the remainder (24). The type III collagen molecule is more flexible than the type I collagen molecule; thus, fibers rich in type III collagen are more extensible than those rich in type I collagen (i.e., type III collagen is less stiff than type I collagen) (25, 26). In other words, the number of fibroblasts and the procollagen secreted by the two types are important clues to explore the cause of POP. Therefore, the stimulation of E2 can ultimately enhance the elasticity and ductility of ligaments. However, cell proliferation without estrogen stimulation is weaker, and procollagen expression is lower, which may explain why POP is more serious in postmenopausal women. Analyzing the pathogenesis of POP from the perspective of fibroblast-procollagen production source is an innovative point of the present study as well.

In the current research, when estrogen was used to stimulate cells, Mfn2 expression was inhibited at both protein level and mRNA level, indicating that estrogen can play a role by inhibiting Mfn2 expression. Mfn2 is a protein that crosses the mitochondrial bilayer membrane twice (8–11). It is not only related to the fusion and division of mitochondria, but also cell proliferation, apoptosis, and signal transduction pathway (8–11). Additionally, Mfn2 is also related to aging (12–14). Our previous *in vivo* and *in vitro* research revealed the relationship between Mfn2 and aging-related diseases; it was unveiled that compared with non-POP patients, Mfn2 has a higher expression in uterosacral ligament fibroblasts of POP patients, and Mfn2 can reduce the supporting effect of ligament and promote the occurrence and development of POP *via* inhibiting the proliferation of fibroblasts and the secretion of procollagen (15–17). The results of the present study further supported our previous results that Mfn2 may play a pivotal role in the occurrence and development of POP. However, estrogen may improve the symptoms of POP by inhibiting the expression level of Mfn2, promoting the proliferation of fibroblasts and the synthesis of procollagen. However, a limited number of reports concentrated on the relationship between Mfn2, estrogen, and POP, which is also a novelty of the present study.

At present, the interaction between Mfn2 and E2 is not clear. Some scholars have confirmed that (27) E2 can inhibit the proliferation of breast cancer cells by inhibiting the expression of Mfn2. Denardo et al. (28) identified a group of estrogen induced genes, including Mfn2. In our study, when we stimulated POP fibroblasts with E2, Mfn2 decreased and cell proliferation increased. These were consistent with the results of previous study. According to traditional studies (29–31), functions of E2 were mainly mediated by receptors, including nuclear receptors (including ERα and ERβ) and membrane receptor GPR30 (GPER-1). After binding with the receptor, E2 activated the transcription and translation of specific genes, and synthesized new functional proteins, thus producing biological effects. There are relatively few studies on the relationship between these receptors and Mfn2. For example, ERα transcription factor was a key regulator of Mfn2 transcription (29). It has been found that a specific region of Mfn2 promoter could bind to and be activated by ERα (30). In addition, E2/GPER/ERK pathway could promote cell proliferation by up-regulating cyclinD1 (31). So far, the relationship between ERβ and Mfn2 is not clear. In this study, we found that the mRNA and protein expression of three receptors increased after E2 stimulation. Therefore, we speculated that the interaction between these three receptors and Mfn2 might play an important role in the effect of E2 on fibroblasts from POP patients. We will continue to explore the mechanism of their interaction in the follow-up study.

The researchers believed that (32, 33) E2 could play a protective role by regulating oxidative stress and preventing mitochondrial dysfunction, leading to the reduction of caspase activation and inhibition of apoptosis. In this project, we used AO/EB and flow cytometry to detect the level of apoptosis. However, there was no significant difference in apoptosis among these groups. These indicated that E2 did not affect the apoptosis of primary fibroblasts from POP patients under the concentration gradient and time gradient set in this study.

We, in the current research, found that only 10^-8^ mol/L of estrogen can significantly change the function of cells, while higher concentrations (10^-6^ and 10^-7^ mol/L) of E2 are not satisfactory, indicating that for *in vitro* tests, higher concentrations of estrogen are not superior. The 10^-8^ mol/L concentration of estrogen is also the physiological concentration (34). In clinical practice, the influence of estrogen concentration in blood on women’s health is highly significant. Especially for postmenopausal women, high estrogen level may increase the risk of some diseases, such as breast cancer, endometrial cancer, Alzheimer’s disease, diabetes, cardiovascular disease, etc. (34–37). For instance, People (34) found that high estrogen levels are independent predictors of dementia, especially in postmenopausal diabetic women. Additionally, a number of scholars demonstrated that (37) the health characteristics of senior females who use estrogen alone or in form of long-term combination of estrogen and progesterone are more pronounced than those who have never used them. This suggests that in terms of drug safety, in order to prevent or control the progress of POP and avoid the high blood concentration caused by systemic drug use, clinicians may further pay attention to local medication. How to control estrogen in blood at a reasonable level, not only to control POP, but also not to cause other diseases, remains to be further studied. According to the results of different time gradients, it can be concluded that the results of 72 and 96 h *in vitro* test are more significant than those of 24 and 48 h, indicating that it is reasonable to recommend the application of E2 once or twice a week.

## Conclusions

In conclusion, the use of estrogen possesses certain benefits for the function of uterosacral ligament fibroblasts in POP patients. Estrogen can promote the expression of downstream procollagen and the proliferation of fibroblasts by inhibiting the expression level of Mfn2 in uterosacral ligament fibroblasts. This effect is time and concentration-dependent (the effect is the greatest when estrogen concentration is 10^-8^ mol/L for 72 and 96 h). The limitation of the present study was that the sample size was extremely small and the observation time after estrogen stimulation was not long enough, requiring further improvement. However, in order to avoid the interference of estrogen in serum, we used serum-free medium for cell culture. After being treated with E2 for more than 96 h (e.g. 120 h or more), the cells were in relatively poor condition and they were no longer suitable for objective analysis of their proliferation, apoptosis and protein expression. In addition, the interaction between E2, Mfn2, and ERs, GPR30 needs to be further improved in future research. In addition, we will further verify our findings through animal experiments.

## Data Availability Statement

The raw data supporting the conclusions of this article will be made available by the authors, without undue reservation.

## Ethics Statement

The studies involving human participants were reviewed and approved by the Ethics Committee of Peking University First Hospital Approval No. 2016(1173). The patients/participants provided their written informed consent to participate in this study.

## Author Contributions

Conception, design and obtaining funding, YL. Drafting of the manuscript, X-QW. Analysis and interpretation of data, X-QW and B-BX. Communicating with patients, R-JH. Critical revision of the manuscript for important intellectual content, YL. All authors contributed to the article and approved the submitted version.

## Funding

The present study was supported by a grant from the Beijing Natural Science Foundation (grant No. 7182167) and the National Natural Science Foundation of China NSFC (grant No. 81401185).

## Conflict of Interest

The authors declare that the research was conducted in the absence of any commercial or financial relationships that could be construed as a potential conflict of interest.
